# Treating the Intestine with Oral ApoA-I Mimetic Tg6F Reduces Tumor Burden in Mouse Models of Metastatic Lung Cancer

**DOI:** 10.1038/s41598-018-26755-0

**Published:** 2018-06-13

**Authors:** Arnab Chattopadhyay, Xinying Yang, Pallavi Mukherjee, Dawoud Sulaiman, Hannah R. Fogelman, Victor Grijalva, Steven Dubinett, Tonya C. Wasler, Manash K. Paul, Ramin Salehi-Rad, Julia J. Mack, M. Luisa Iruela-Arispe, Mohamad Navab, Alan M. Fogelman, Srinivasa T. Reddy

**Affiliations:** 1 Department of Medicine, David Geffen School of Medicine, University of California, Los Angeles, CA 90095-1736 USA; 2 Department of Obstetrics and Gynecology, David Geffen School of Medicine, University of California, Los Angeles, CA 90095-1736 USA; 3 Molecular Toxicology Interdepartmental Degree Program, Fielding School of Public Health, University of California, Los Angeles, CA 90095-1736 USA; 4 Department of Molecular, Cell and Developmental Biology, College of Letters and Science, University of California, Los Angeles, CA 90095-1736 USA; 5 Department of Molecular and Medical Pharmacology, David Geffen School of Medicine, University of California, Los Angeles, CA 90095-1736 USA

## Abstract

Having demonstrated that apolipoprotein A-I (apoA-I) mimetic peptides ameliorate cancer in mouse models, we sought to determine the mechanism for the anti-tumorigenic function of these peptides. CT-26 cells (colon cancer cells that implant and grow into tumors in the lungs) were injected into wild-type BALB/c mice. The day after injection, mice were either continued on chow or switched to chow containing 0.06% of a concentrate of transgenic tomatoes expressing the apoA-I mimetic peptide 6F (Tg6F). After four weeks, the number of lung tumors was significantly lower in Tg6F-fed mice. Gene expression array analyses of jejunum and lung identified Notch pathway genes significantly upregulated, whereas osteopontin (Spp1) was significantly downregulated by Tg6F in both jejunum and lung. In jejunum, Tg6F increased protein levels for Notch1, Notch2, Dll1, and Dll4. In lung, Tg6F increased protein levels for Notch1 and Dll4 and decreased Spp1. Tg6F reduced oxidized phospholipid levels (E06 immunoreactivity) and reduced 25-hydroxycholesterol (25-OHC) levels, which are known to inhibit Notch1 and induce Spp1, respectively. Notch pathway promotes anti-tumorigenic patrolling monocytes, while Spp1 facilitates pro-tumorigenic myeloid derived suppressor cells (MDSCs) formation. Tg6F-fed mice had higher numbers of patrolling monocytes in jejunum and in lung (p < 0.02), and lower plasma levels of Spp1 with reduced numbers of MDSCs in jejunum and in lung (p < 0.03). We conclude that Tg6F alters levels of specific oxidized lipids and 25-OHC to modulate Notch pathways and Spp1, which alter small intestine immune cells, leading to similar changes in lung that reduce tumor burden.

## Introduction

There is both epidemiologic data and data from animal models, which suggest a possible role in cancer for high-density lipoproteins (HDL) and the main protein in HDL, apolipoprotein A-I (apoA-I). Jafri *et al*.^1^ reported that in 24 randomized clinical trials with 625,477 person-years of follow-up and 8,185 incident cancers, there was a significant inverse association between the baseline HDL-cholesterol level and rate of incident cancer. The inverse association persisted after adjusting for baseline low density lipoprotein (LDL)-cholesterol, age, body mass index, diabetes, gender, and smoking status^1^. For every 10-mg/dL increment in HDL-cholesterol there was a 36% relatively lower rate of the development of cancer (p < 0.001)^1^. Borgquist *et al*.^2^ reported that in a population-based prospective cohort study with 17,035 women and 11,063 men in which incident cancer cases were ascertained by record linkage with the Swedish Cancer Registry, the baseline levels of apoA-I, were inversely associated with lung cancer risk in both genders. Su *et al*.^3^ demonstrated that apoA-I, and small peptide mimetics of apoA-I were effective in inhibiting tumor development in a mouse model of ovarian cancer. Subsequently, this group reported that an apoA-I mimetic peptide inhibited tumor angiogenesis by suppressing VEGF/basic FGF signaling pathways^4^. The upregulation of the antioxidant enzyme manganese superoxide dismutase (MnSOD) by an apoA-I mimetic peptide was also shown to contribute to the inhibition of proliferation and tumorigenicity of epithelial ovarian cancer cells^5^. Peptides mimicking the proteins associated with HDL were also shown to inhibit tumor development in both induced and spontaneous mouse models of colon cancer^6^. Gao *et al*.^7^ reported that apoA-I mimetic peptides inhibit the expression of hypoxia-inducible factor-1α (HIF-1α) in human ovarian cancer cell lines and in a mouse ovarian cancer model. Yang *et al*.^8^ found that biomimetic synthetic HDL nanostructures targeted scavenger receptor type B-1 and induced apoptosis in B-cell lymphoma in mice. Neyen *et al*.^9,10^ reported that an apoA-I mimetic targets scavenger receptor A on tumor-associated macrophages and inhibited tumor progression and metastasis. Zamanian-Daryoush *et al*.^11^ reported that apoA-I suppresses tumor growth and metastasis in multiple animal tumor models including the aggressive B16 F10L murine malignant melanoma model. The mechanism of action appeared to be related to the ability of apoA-I to induce infiltration of CD11b^+^ F4/80^+^ macrophages with an M1 anti-tumor phenotype, and also to decrease the number of myeloid-derived suppressor cells (MDSCs)^11^. Cedo *et al*.^12^ reported that an apoA-I mimetic peptide, but not apoA-I itself inhibited tumor growth in a mouse model of inherited breast cancer.

Our previous work^13^ provided further evidence that apoA-I and apoA-I mimetic peptides ameliorate cancer in mouse models by immunomodulatory mechanisms in which a model of colon cancer metastatic to the lungs in wild-type BALB/c mice was used. After injection of colon cancer cells into the tail vein of the mice, the mice were continued on standard mouse chow or were switched to standard mouse chow supplemented with a concentrate of transgenic tomatoes expressing the apoA-I mimetic peptide 6F (Tg6F) added at 0.06% by weight of the diet. The mice receiving Tg6F demonstrated a two-thirds reduction in lung tumor burden that was not seen in mice given the same dose of concentrate from control tomatoes that expressed a marker protein instead of the 6F peptide^13^. Remarkably, the mice receiving the Tg6F concentrate in their diet had a 94 ± 1.1% reduction in tumor-associated neutrophils^13^. Since the 6F peptide was found in the small intestine after feeding, but was not detected in the plasma^14^, it was assumed that Tg6F must have modulated the immune system in the small intestine resulting in an altered immune response in the lung to the injected tumor cells^13^.

Here we report on two mouse models of metastatic lung cancer, i) BALB/c mice injected intravenously with colon cancer cells that metastasize to the lungs; and ii) C57BL/6J mice injected with Lewis lung cancer cells that metastasize to the lungs. In each case switching the diet to chow supplemented with 0.06% of Tg6F starting on the day after the injection of the cancer cells resulted in a dramatic decrease in tumor burden. Switching to the diet containing Tg6F resulted in changes in cytokine expression that were similar in the lamina propria of the small intestine and in the lung. Cancer pathway gene arrays followed by validation experiments identified *Notch*1 *and Osteopontin (Spp1)* to be differentially regulated by Tg6F in both the intestine and lung. Oral administration of Tg6F reduces both levels of oxidized phospholipids recognized by the natural antibody, E06, and 25-hydroxycholesterol (25-OHC) levels, which are known to effect Notch1 and Spp1, respectively. Oral administration of Tg6F also resulted in an increase in patrolling monocytes in the lamina propria of the villi of the small intestine, and in the lung, that was similar in magnitude. Patrolling monocytes have previously been reported to be associated with inhibition of tumor metastasis to the lungs^15^. Additionally, feeding Tg6F resulted in a decrease in myeloid derived suppressor cells (MDSCs) in the lamina propria of the small intestine and in the lung, that was similar in magnitude. MDSCs have previously been reported to promote tumor growth^11^. Recent studies have shown that the Notch pathway promotes patrolling monocytes^16^ whereas Spp1 facilitates MDSC formation^17,18^. These studies suggest that oral administration of an apoA-I mimetic peptide alters specific oxidized lipids, which alter cytokines and immune cells in the lamina propria of the small intestine resulting in favorable immunomodulation of cancers in tissues distant from the intestine.

## Results

### Tg6F treatment reduced lung tumor burden in a mouse model of colon cancer metastatic to lung

**Figure 1:**
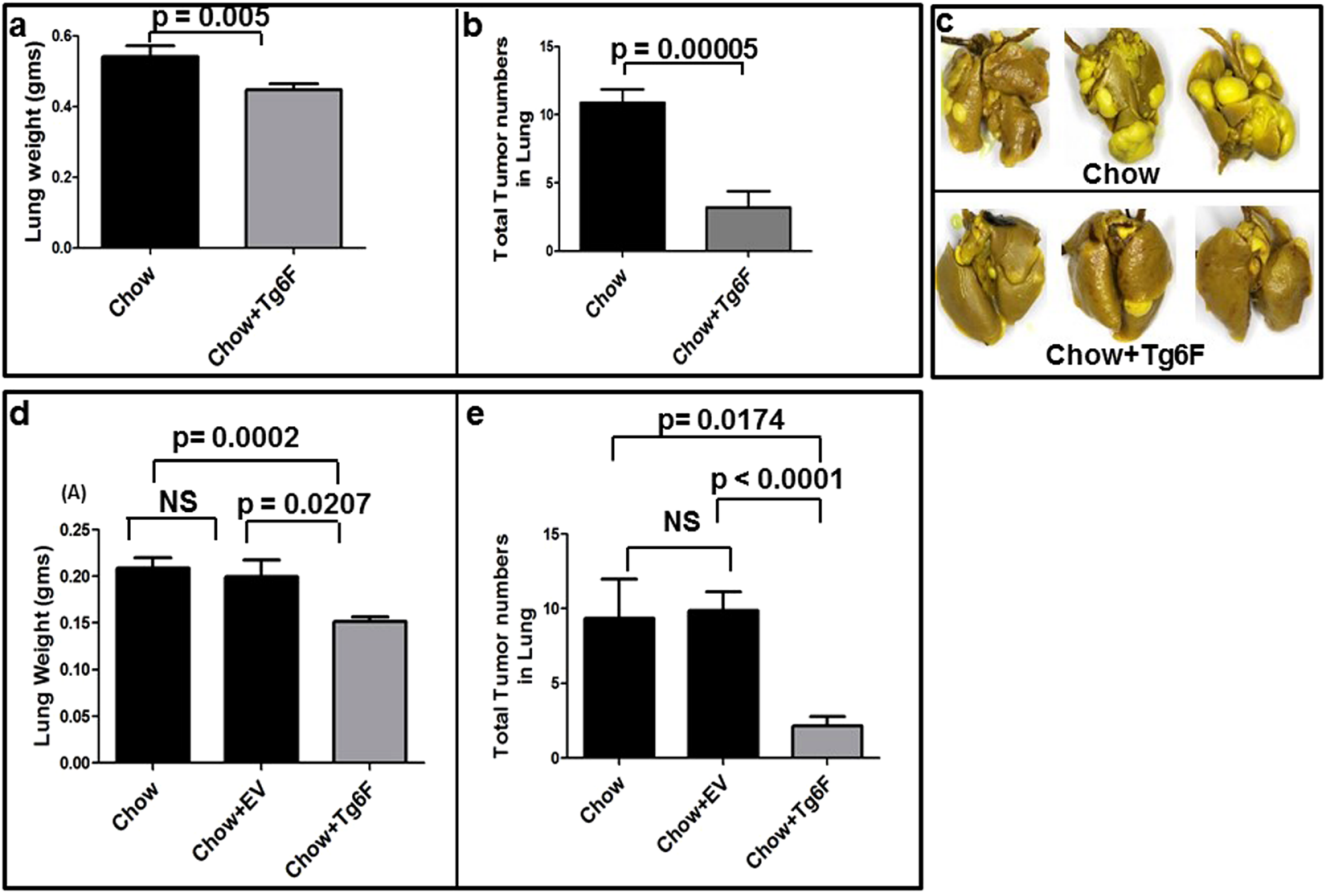
Tg6F decreased lung weight and the number of lung tumors in mice administered CT26 colon cancer cells. Female BALB/c mice 6 weeks of age (n = 10 per group) were injected with 2 × 10^4^ CT26 cells in 100 μL of PBS via tail vein. The day after injection, the mice were either maintained on standard mouse chow (Chow), or were switched to standard mouse chow containing 0.06% by weight of the transgenic 6F tomato concentrate (Tg6F). After 4 weeks, the mice were subjected to a terminal bleed, and after sacrifice the lungs were harvested, weighed, and fixed with Bouin’s solution, and the number of tumor nodules on the surface of the lungs was determined as described in Materials and Methods. (**a**) Lung weight. (**b**) Number of tumor nodules on the surface of the lungs. (**c**) Representative pictures of lungs with tumors from mice in (**d,e**). Tg6F treatment reduces tumor burden in Lewis lung carcinoma mouse model of lung cancer. Female C57BL/6J mice 2 months of age (n = 12 per group) were administered 0.15 × 10^6^ 3LL cells *via* tail vein injection. The day after injection the mice were maintained on mouse chow or were switched to mouse chow containing 0.06% by weight of the control tomato concentrate (EV) or the concentrate containing the 6F peptide (Tg6F). After one month the lungs were harvested, weighed, fixed and the number of tumors on the surface of the lungs was determined as described in Materials and Methods. (**d**) Lung weight. (**e**) Number of tumor nodules on the surface of the lungs. The data shown are Mean ± SEM. The data shown are Mean ± SEM. NS = Not significant.

Confirming our previous report^13^, adding 0.06% by weight of Tg6F to the chow of wild-type BALB/c mice the day after intravenous administration of CT26 colon cancer cells, and continuing the diet for 4 weeks, resulted in a significant reduction in lung weight (Fig. 1a). There was no change in body weight (data not shown), but there was a dramatic (~75% reduction) in total tumor nodules (Fig. 1b,c) compared to mice who continued on chow alone.

### Tg6F treatment reduced lung tumor burden in Lewis lung carcinoma mouse model of lung cancer

Figure 1d,e show that Tg6F treatment of wild-type C57BL/6J mice after intravenous administration of Lewis lung carcinoma (3LL) cells significantly reduced lung weight and the number of tumors found on the surface of the lungs. The data in Fig. 1d,e also show that the control transgenic tomato concentrate (EV) was not effective, strongly suggesting that the effectiveness of Tg6F is due to the presence of the 6F peptide, which is not present in EV.

### Tg6F treatment changed the expression of cytokines and inflammatory genes similarly in the small intestine and lung

**Figure 2:**
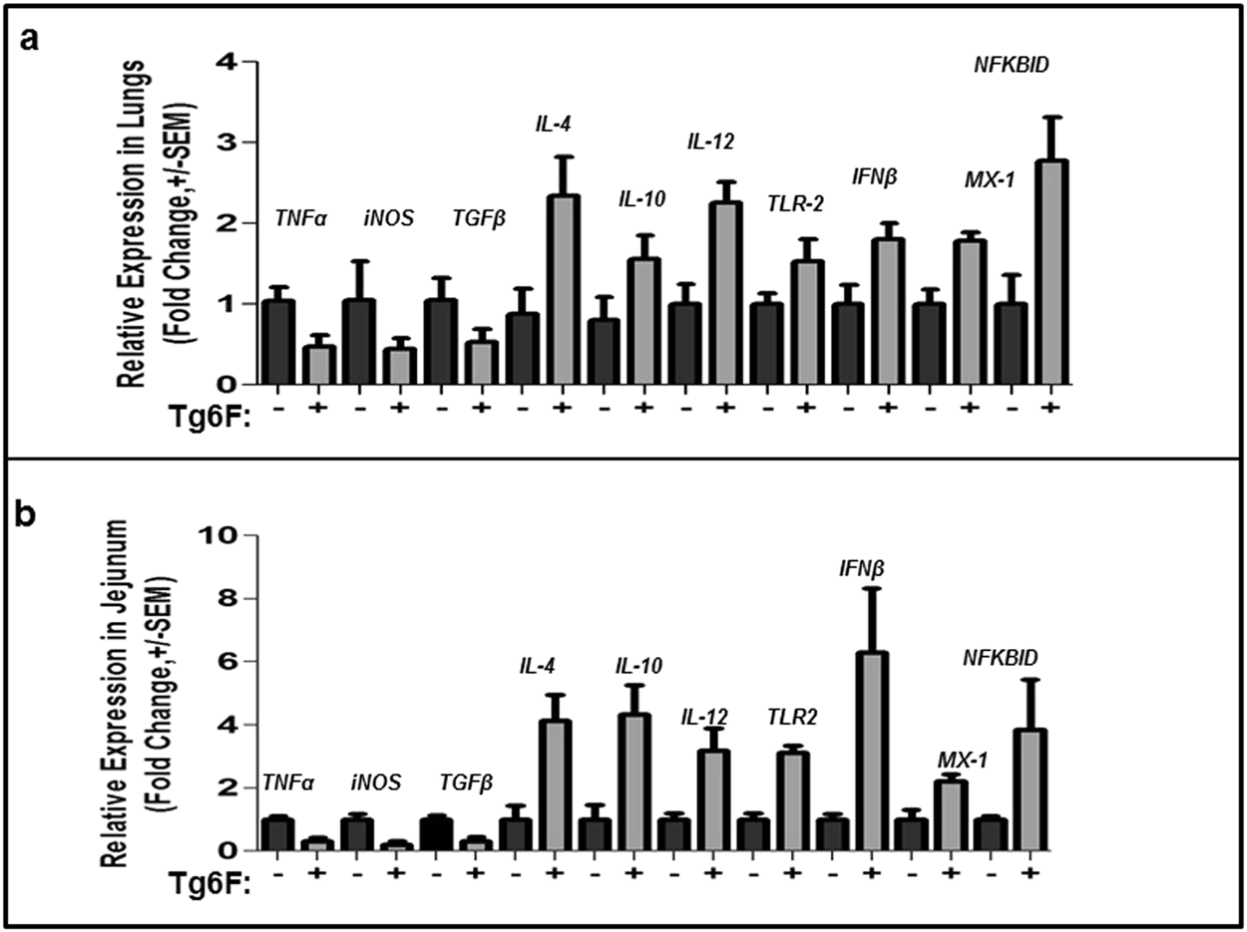
Tg6F changes the expression of genes similarly in the jejunum and lung. RNA was isolated from the jejunum or the lungs of the BALB/c mice described in Fig. 1 and gene expression of *TNFα, iNOS, TGFβ,IL-4,IL-10,IL-12,TLR-2, IFNβ, MX-1,NFKBID* was determined by RT-qPCR as described in Materials and Methods in (**a**) lungs and (**b**) jejunum. The data shown are Mean ± SEM.

Adding 0.06% by weight of Tg6F to mouse chow the day after intravenous administration of CT26 colon cancer cells, and continuing the diet for 4 weeks resulted in a similar change in expression of several genes in the jejunum and lung. The Tg6F-fed mice expressed lower levels of *TNFα*, *iNOS*, and *TGFβ* compared to chow-fed mice, in both jejunum and lung (Fig. 2a,b). The Tg6F-fed mice expressed higher levels of *IL-4*, *IL-10*, *IL-1*2, *TLR2*, *IFNβ*1, *MX1* and *NFKBID* compared to chow-fed mice in both jejunum and lung (Fig. 2a,b).

### Tg6F treatment altered the expression of cancer pathway genes in the small intestine and lung

**Figure 3:**
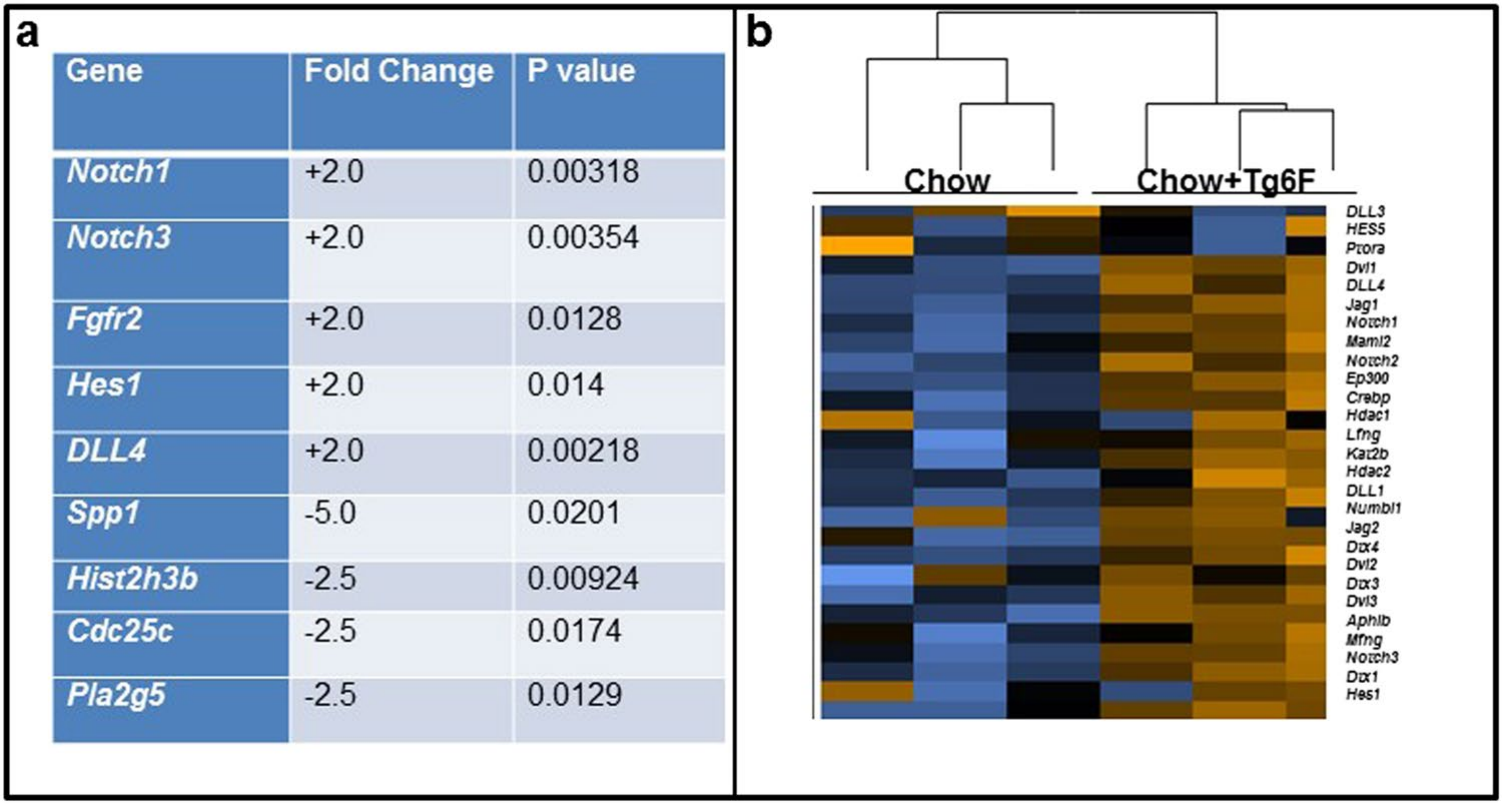
Gene array analysis and expression of Notch pathway genes. Total RNA was isolated from the jejunum or the lungs (n = 3 per group) of the BALB/c mice described in Fig. 1. 100 ng RNA was analyzed by the nCounter PanCancer mouse pathway Panel (NanoString Technologies) containing 770 genes, according to the manufacturer’s protocol as described in Materials and Methods. (**a**) List of select genes differentially expressed in the lungs of control and Tg6F treated mice that were greater than 1.5-fold upregulated or downregulated with a p-value < 0.05. Genes belonging to Notch family were upregulated while others including osteopontin (*Spp1*) were downregulated. (**b**) Heat map of Notch family genes in lung tissue obtained from control and Tg6F groups (n = 3 per group).

To further understand the anti-tumorigenic mechanisms of Tg6F, we performed a cancer pathway gene array analysis (Nanostring Technologies) using multiplex gene expression analysis with 770 genes from 13 cancer-associated canonical pathways. The pathways included: *MAPK*, *STAT*, *PI3K*, *RAS*, *Cell Cycle*, *Apoptosis*, *Hedgehog*, *Wnt*, DNA Damage Control, Transcriptional Regulation, Chromatin Modification, and *TGF-β*. Tg6F administration increased the signature of most of the pathways tested, but the DNA damage repair and cell cycle pathway signatures were lower than in the controls (Supplemental Fig. 1). Differences in gene expression were analyzed at the individual gene level. Tg6F differentially regulated several genes when compared to no treatment. A short list of genes upregulated and downregulated by Tg6F in the lungs is shown in Fig. 3a. Since *Notch* family members were represented in the short list, a heat map was generated for genes in the *Notch* pathway. As shown in Fig. 3b and Supplemental Fig. 2, Tg6F had a dramatic effect on the Notch pathway.

### Tg6F treatment induced the expression of Notch pathway in both the small intestine and the lungs

**Figure 4:**
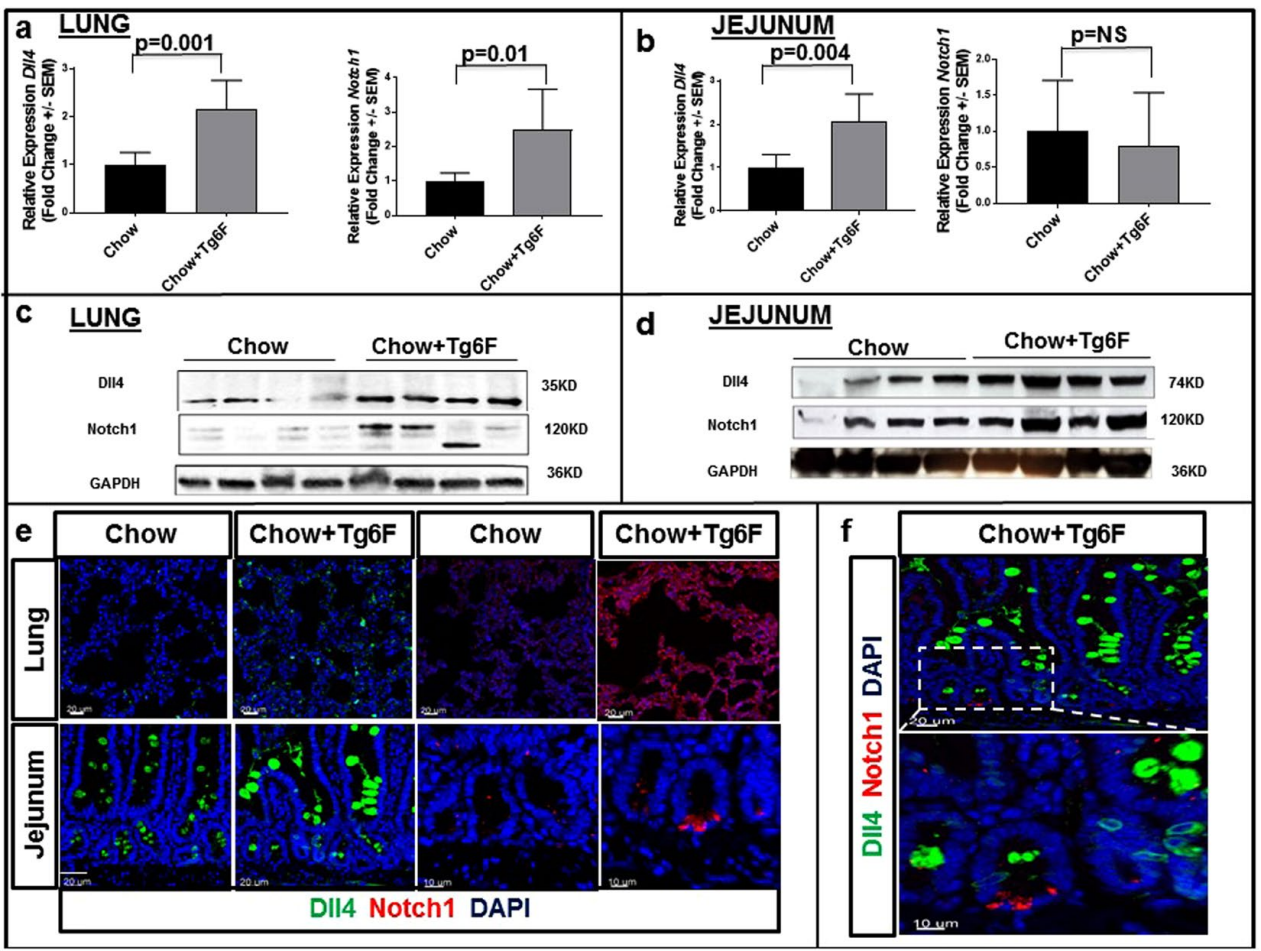
Tg6F induced Dll4 and Notch1 gene expression. RNA was isolated from the jejunum and lungs of the BALB/c mice described in Fig. 1 and gene expression was quantified by RT-qPCR as described in Materials and Methods. Tg6F induced *Dll4* and *Notch1* mRNA expression in the tissue of (**a**) lung and (**b**) in the tissue of jejunum. The data shown are Mean ± SEM. *Tg6F induced* Dll4 *and* Notch1 *protein expression in both lungs and jejunum tissues*. Protein lysates were isolated from tissues of the BALB/c mice described in Fig. 1 and were analyzed by immunoblot for expression of Dll4 and Notch1 in the lung (**c**) and jejunum (**d**). Expression of Notch1 and Dll4 proteins by immunofluorescence. Tissue from lung and jejunum were obtained from the BALB/c mice described in Fig. 1, and analyzed by confocal microscopy for immunofluorescence as described in Materials and Methods. Representative images of sections from single antibody staining of lung and jejunum tissues (**e**) and dual staining of Chow+ Tg6F treated jejunum tissue (**f**) are shown.

**Figure 5:**
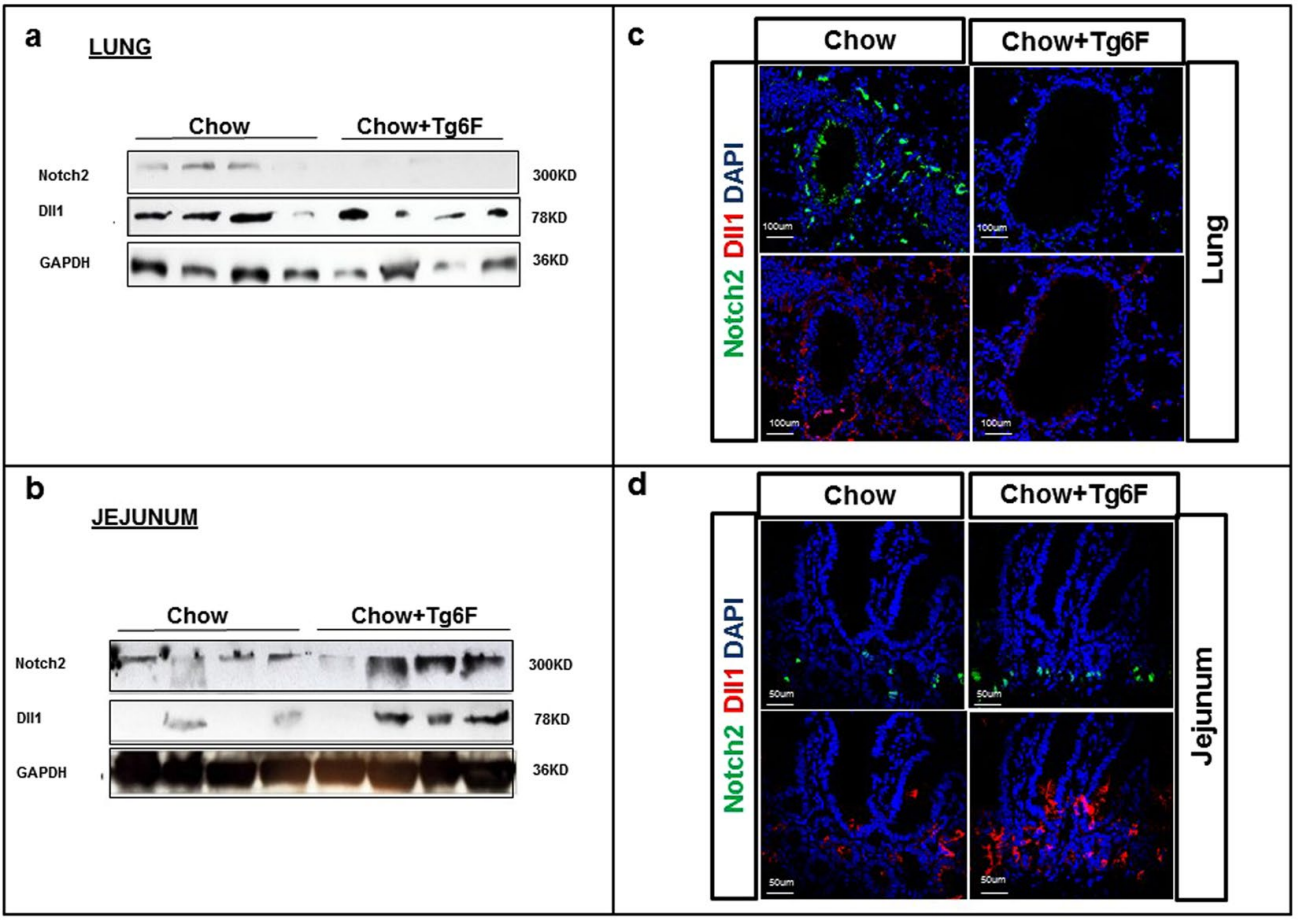
*Tg6F induced* Dll1 *and* Notch2 *protein expression in jejunum tissues but not in the lungs*. Protein lysates from jejunum and lung tissues of the BALB/c mice described in Fig. 1 were analyzed by immunoblot and immunofluorescence for expression of Dll1 and Notch2. Panels (**a**) and (**c**) show Notch2 and Dll1 expression in lung. Panels (**b**) and (**d**) show Notch 2 and Dll1 expression in jejunum.

To validate the gene array data (Fig. 3a,b), we analyzed mRNA and protein expression levels for several members of *Notch* family in the small intestine and lungs obtained from the mice described in Fig. 1. Two Notch family members, Notch1 (protein) and Dll4 (mRNA and protein) were consistently and significantly induced in Tg6F-fed mice (Fig. 4a–f) in both small intestine and lungs when compared to control mice. Although Notch2 and Dll1 mRNAs were not induced by Tg6F in both the small intestine and lungs (data not shown), we observed that both Notch2 and Dll1 proteins were increased by Tg6F in the jejunum *but not* in lung tissue (Fig. 5).

### Tg6F prevented the accumulation of oxidized phospholipids detected by E06 in the small intestine

**Figure 6:**
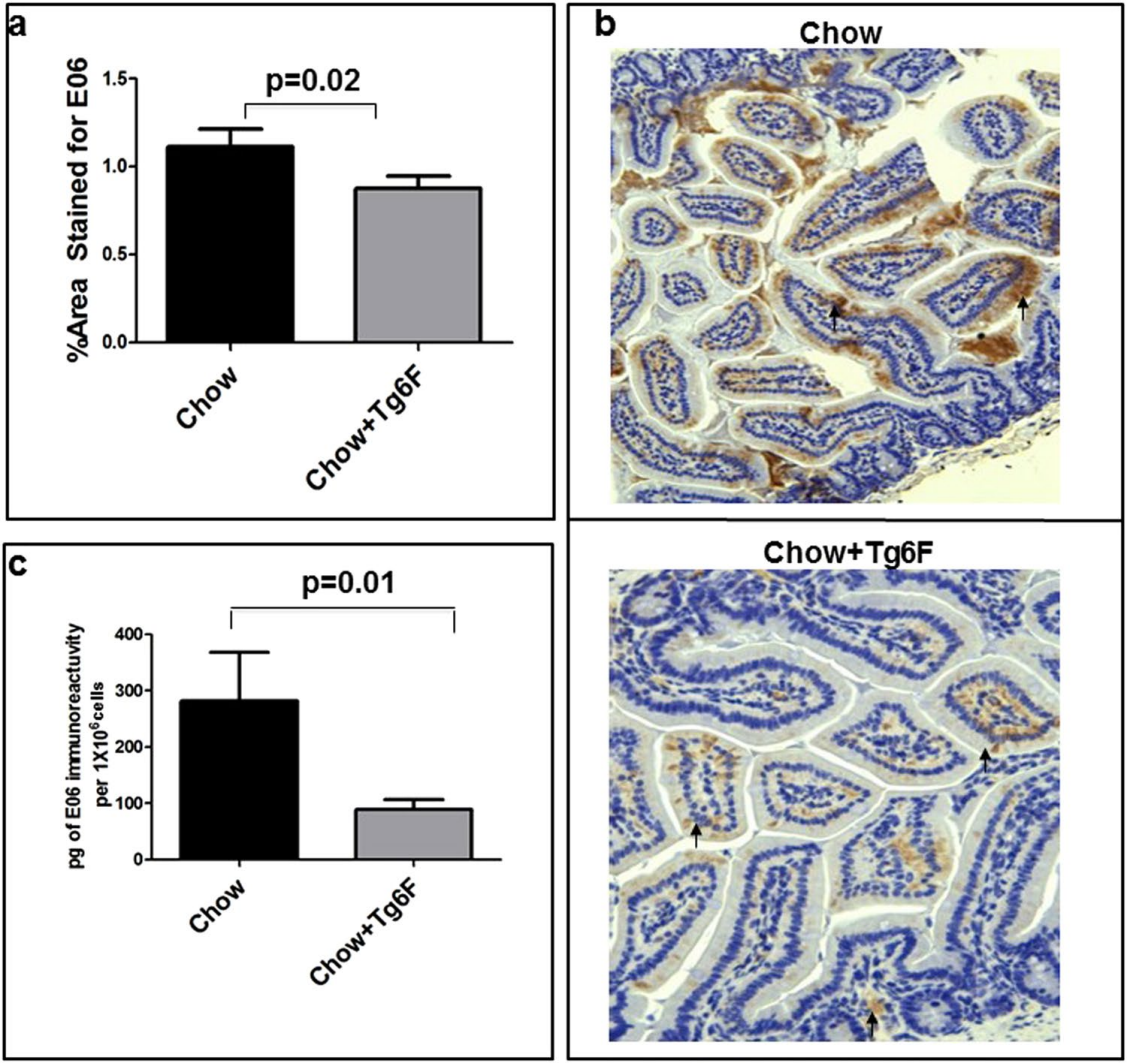
E06 levels in the jejunum were reduced in Tg6F fed mice. Female BALB/c mice 6–7 weeks of age (n = 10 per group) were fed standard mouse chow (Chow), or standard mouse chow containing 0.06% by weight of the transgenic 6F tomato concentrate (Tg6F). After 4 weeks, the mice were fasted overnight the jejunum was harvested, and luminal contents were removed by washing, the jejunum was processed, and enterocytes were prepared and analyzed as described in Materials and Methods. (**a**) Percent area stained for E06 in jejunum. (**b**) Representative photomicrograph of a section of jejunum stained for E06 (brown). (**c**) E06 levels were quantified by ELISA in the enterocyte extracts as described in Materials and Methods. The results shown are Mean ± SEM.

Notch1 expression in the endothelium is inhibited by oxidized phospholipids including oxidized 1-palmitoyl-2-arachidonoyl-sn-glycero-3-phosphocholine (Ox-PAPC)^19^. E06 is a natural antibody that recognizes specific components of Ox-PAPC, and provides a well validated means of measuring the levels of these oxidized phospholipids^20^. Tg6F treatment significantly reduced E06 levels in the jejunum, as shown by immunohistochemistry and in enterocytes shown by an ELISA (Fig. 6).

### Tg6F treatment inhibited the expression of osteopontin(Spp1) in both the small intestine and the lungs

**Figure 7:**
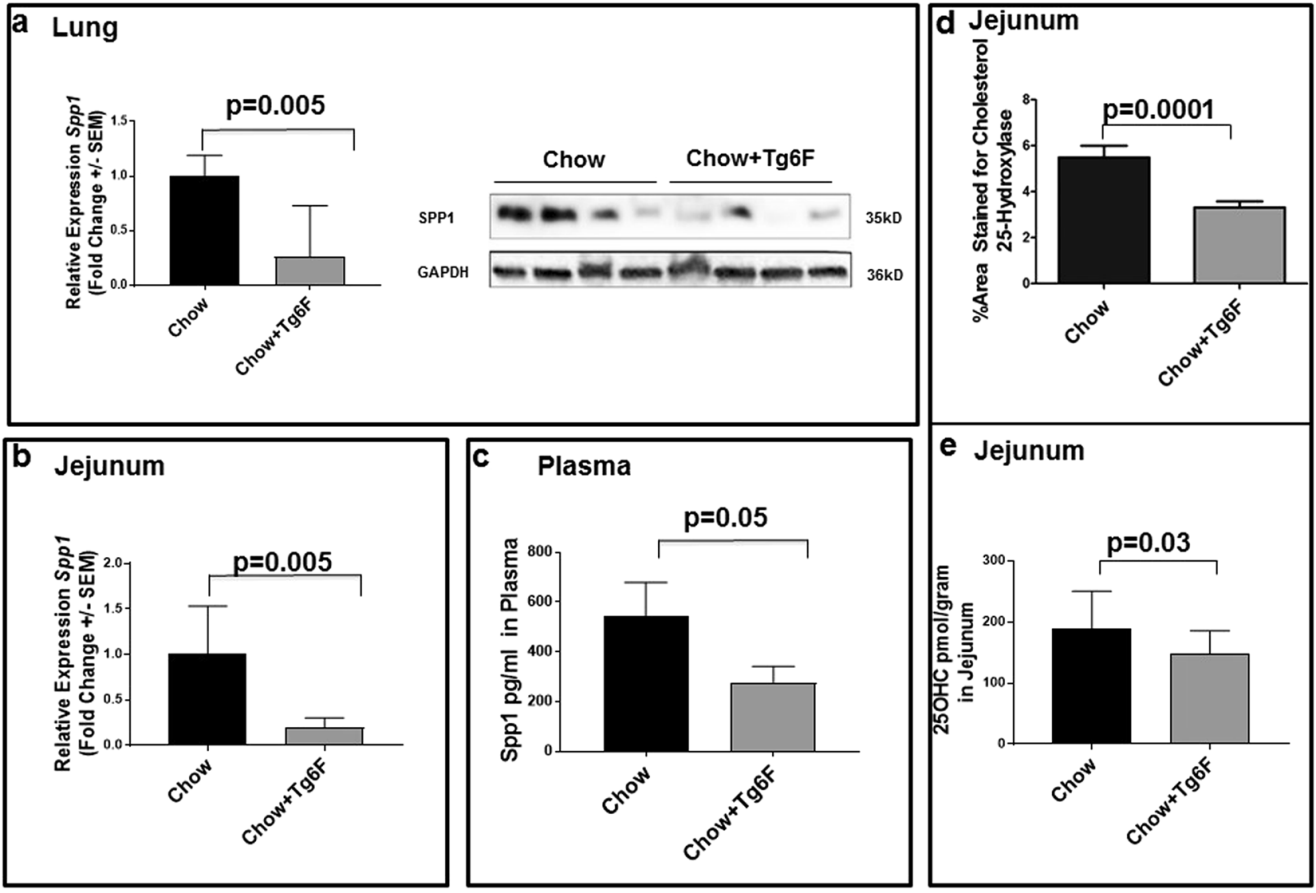
Spp1 (osteopontin) mRNA and Spp1 protein expression were reduced in Tg6F fed mice. Total RNA and protein were isolated from the lungs of the BALB/c mice described in Fig. 1. (**a**) *Spp1* mRNA expression was quantified by RT-qPCR (left side of panel) and protein lysates from lungs were analyzed by immunoblotting for Spp1 (right side of panel) as described in Materials and Methods. Each lane in the immunoblot is from a separate mouse. (**b**) *Spp1 mRNA expression was reduced in the intestines of Tg6F fed mice*. Total RNA was isolated from the jejunum of the BALB/c mice described in Fig. 1. *Spp1* mRNA expression was quantified by RT-qPCR as described in Materials and Methods. The data shown are Mean ± SEM. (**c**) Tg6F decreased Spp1 levels in the plasma as measured by ELISA as described in Materials and Methods. The data shown are Mean ± SEM. (**d**) Cholesterol 25-hydroxylase (CH25H) protein expression in the villi of the jejunum was quantified by immunohistochemistry as described in Materials and Methods. The results shown are Mean ± SEM. (**e**) 25-hydroxycholesterol (25-OHC) levels in jejunum were determined by LC-MS/MS as described in Materials and Methods. The results shown are Mean ± SEM.

*Spp1* is one of the genes that was significantly reduced by Tg6F treatment in the gene array studies (Fig. 3a). To validate the gene array data, we analyzed mRNA and protein expression levels for Spp1 in tissue from the lungs and small intestine obtained from the mice described in Fig. 1. Tg6F significantly reduced the levels of Spp1 in the lungs (Fig. 7a), jejunum (Fig. 7b), and plasma (Fig. 7c).

### Tg6F reduced levels of cholesterol 25-hydroxylase (CH25H) in jejunum and 25-hydroxy cholesterol (25-OHC) in plasma

Oxysterols including 25-OHC induce osteopontin (Spp1) expression^21^. We recently demonstrated that Tg6F treatment inhibited CH25H and 25-OHC in LDLR null mice fed a Western Diet^22^. Tg6F treatment in the cancer model inhibited the expression of CH25H as measured by IHC in jejunum (Fig. 7d and Supplemental Fig. 3), and decreased the product of CH25H activity (25-OHC) in jejunum (Fig. 7e).

### Tg6F changed the composition of immune cells similarly in the small intestine and lungs

**Figure 8:**
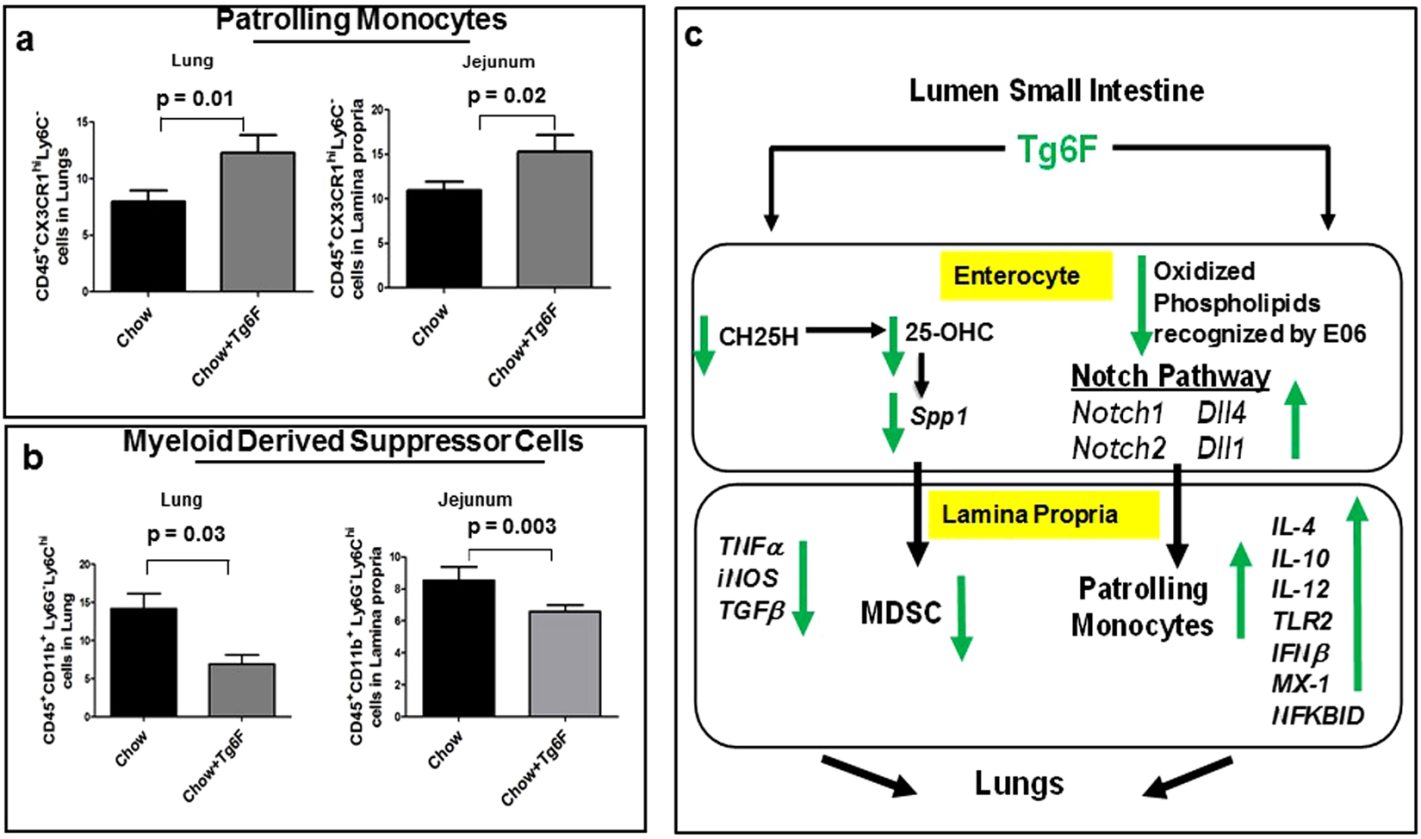
Tg6F changed the composition of immune cells similarly in the small intestine and lungs providing a working hypothesis/model for the anti-tumorigenic activity of Tg6F in mice. Immune cells were isolated from the lamina propria of the jejunum or from the lungs as described in Materials and Methods and were sorted by flow cytometry for patrolling monocytes or myeloid derived suppressor cells as described in Materials and Methods. (**a**) Quantification of patrolling monocytes in the lung and in the lamina propria of the jejunum. The results shown are Mean ± SEM. (**b**) Quantification of myeloid derived suppressor cells in the lung and in the lamina propria of the jejunum. The data shown are Mean ± SEM. (**c**) Working hypothesis/model for the anti-tumorigenic activity of Tg6F in mice. Tg6F decreases the expression of cholesterol 25-hydroxylase (CH25H) which leads to a decrease in levels of 25-hydroxycholesterol (25-OHC), which in turns leads to decreased levels of osteopontin (Spp1), which leads to decreased levels of myeloid derived suppressor cells (MDSC) in the small intestine. Tg6F also decreases the levels of oxidized phospholipids recognized by the E06 antibody, which leads to increased activity of the Notch pathway with increased levels of Notch1 and Dll4 and Notch2 and Dll1 that increase levels of patrolling monocytes in the small intestine. Additionally, Tg6F alters the expression of cytokines and factors involved in modulating inflammation; TNFα, iNOS and TGFβ expression is decreased, while IL-4, IL-10, IL-12, TLR2, IFNβ, MX-1 and NFKBID expression is increased in the small intestine. The results of these changes in the small intestine are transmitted to the lungs where similar changes occur and result in decreased tumor burden in the lungs following injection of malignant cells into the tail vein of mice with normal immune systems.

Gamrekelashvili *et al*.^16^ reported that the Notch pathway plays an important role in regulating the level of patrolling monocytes in the vasculature. We hypothesized that the Tg6F-mediated increase in *Notch1* and *Notch 2 with increased* Dll4 and Dll1 expression in the jejunum in the cancer model might affect the immune cell repertoire. Adding 0.06% by weight of Tg6F to mouse chow the day after intravenous administration of CT26 colon cancer cells, and continuing the diet for 4 weeks, resulted in a similar change in the composition of the immune cells in the jejunum and lung. An example of the flow cytometry data for patrolling monocytes isolated from the lamina propria of the jejunum is shown in Supplemental Fig. 4. Quantification of the patrolling monocytes (CD45^+^CX3CR1^+^Ly6C^−^) isolated from the lamina propria of the jejunum and the lung are shown in Fig. 8a, and demonstrate similar increases in both small intestine and lung. It has been reported that Spp1 facilitates the fate and function of MDSC^17,18^. Since Tg6F inhibited *Spp1* expression and resulted in lower levels of plasma Spp1 protein, we hypothesized that Tg6F may alter the MDSC population (CD45^+^CD11b^+^Ly6G^−^Ly6C^hi^). An example of the flow cytometry data for MDSC isolated from the lamina propria of the jejunum is shown in Supplemental Fig. 5. Quantification of MDSC isolated from the lamina propria of the jejunum and the lung are shown in Fig. 8b, and demonstrate decreases in both small intestine and lung, but with a somewhat larger decrease in the lung.

## Discussion

The data reported here (Fig. 1a–c) confirm our previous observations^13^ that switching to a chow diet supplemented with 0.06% Tg6F on the day after intravenous injection of CT26 cells into BALB/c mice with a normal immune system resulted in a dramatic decrease in tumor burden in the lungs compared to mice continued on chow alone. The data in Fig. 1d,e demonstrate that the same was true following intravenous injection of 3LL cells into C57BL/6J mice with a normal immune system. These studies confirm that the component of Tg6F accounting for the beneficial effects on tumor growth is the 6F peptide, since the concentrate from transgenic tomatoes lacking the 6F peptide (EV) had no effect on tumor growth (Fig. 1e).

We previously reported that 2 hours after feeding Tg6F, intact peptide was detected in the small intestine, but was below the level of detection in the blood^14^. A peptide whose structure is closely related to 6F (the 4F peptide) was found to have a remarkable affinity for the proximal small intestine (duodenum and jejunum)^23^. One hour after injection into the tail vein of the 4F peptide in C57BL/6 mice, more peptide was found in the small intestine than in the liver^23^. These data together with our prior publications on the 4F peptide^24,25^ and the published data from a peptide structurally unrelated to the 4F or 6F peptides^26,27^ indicate that apoA-I mimetic peptides can act in the intestine to alter systemic inflammation, even in the absence of detectable blood peptide levels.

Feeding a high-fat high-cholesterol diet to either wild-type C57BL/6J mice or C57BL/6J mice lacking the LDL receptor resulted in a significant increase in immune cells in the lamina propria of the jejunum that was ameliorated by adding Tg6F to the diet^28^. None of the mice in the present studies were fed a high-fat high-cholesterol diet. All of the studies reported here were carried out in mice fed standard mouse chow. In contrast to the case for the high-fat high-cholesterol diet where there was a significant decrease in plasma total cholesterol and triglyceride levels after addition of Tg6F to the diet, in the studies reported here, addition of Tg6F to the chow diet did not significantly alter plasma total cholesterol or triglycerides (data not shown).

The role of cytokines and immune cells in cancer metastasis and progression is complex, but there is abundant data indicating that these factors play an important role in these processes^29–31^. The recruitment of immunosuppressive cells protects metastatic cancer cells and promotes tumor growth at these sites^31,32^. MDSCs have been reported to play a role in promoting the metastasis and growth of a number of cancers and have been thought to be the reason for the failure of a number of different cancer treatments^11,33–42^. There are two subtypes of MDSCs, granulocytic and monocytic^43–45^. The two subtypes use different pathways to suppress T cells. The granulocytic MDSCs express high levels of reactive oxygen species, but little nitric oxide, while the monocytic subset produces little reactive oxygen species but high levels of nitric oxide^43^. Nonetheless, the ability to inhibit T cells is not significantly different between these subsets of MDSCs^43^. The expansion of the subsets varies in mice according to the tumor model and the genetic background of the mice^43–45^. The data in Fig. 8b are for monocytic MDSCs and show that Tg6F addition to mouse chow significantly decreased this subset in the lamina propria of the jejunum and in the lung. We did not see any difference in the granulocytic subset of MDSCs in the lamina propria of the jejunum or in the lung after addition of Tg6F (data not shown).

While MDSCs promote metastasis and tumor growth, Hanna *et al*.^15^ demonstrated that a subset of monocytes known as patrolling monocytes (CX3CR1^high^Ly6C^−^ in mice) decrease metastasis to the lung. Patrolling monocytes likely do not directly kill tumor cells; more likely, they recruit NK cells, which kill the tumor cells^15,46^. In the studies by Hanna *et al*.^15^, the rapidity of recruitment of patrolling monocytes to the site of tumor injection was impressive (within 4 hours). The data in Fig. 8a show that after addition of Tg6F to the diet, there was a significant and similar increase in patrolling monocytes in the lamina propria of the jejunum and in the lung. It seems likely that the combined effect of the Tg6F mediated *decrease* in MDSCs and the Tg6F mediated *increase* in patrolling monocytes plays a role in mediating the significant decrease in lung tumor burden (Fig. 1).

We think that it is most likely that the 6F peptide in Tg6F directly acts on the small intestine to alter the cytokine profile (Fig. 2) and immune cells (Fig. 8), which leads to similar changes in the lung. However, in this study, we did not determine the sequence of events (e.g. the time course of changes in the small intestine versus the lung). Future research will be needed to establish the sequence of events and the specific mediators of this intestine-lung axis. However, we have identified the molecules affected by Tg6F that mediate the Tg6F-dependent changes in gene expression. We think it likely that oxidized phospholipids recognized by E06 (Fig. 6) and 25-OHC (Fig. 7) are the specific mediators that were altered by Tg6F leading to the changes in gene expression and immune cell composition in the small intestine and in the lung.

Notch has been implicated as both an oncogene or a tumor suppressor depending on the cell context^47,48^. George *et al*.^49^ identified inactivating mutations in the *Notch* family genes in 25% of human small cell lung cancers (SCLC) and the authors further demonstrated that activation of Notch signaling in a pre-clinical SCLC mouse model inhibited tumors and improved survival^50^. Our results demonstrated that *Notch1* and *Dll4* were induced in Tg6F treated mice in both the small intestine and the lungs, whereas Notch2 and Dll1 were induced in Tg6F treated mice only in the small intestine (Figs 4 and 5). Based on the work of Gamrekelashvili *et al*.^16^, it seems likely that increased Notch signaling altered the fate of naïve monocytes resulting in the increased levels of patrolling monocytes that were observed in Tg6F-treated mice (Fig. 8a). Gamrekelashvili *et al*.^16^ demonstrated that endothelial Notch2 and Dll1 signaling promoted patrolling monocyte differentiation. Our results are consistent with those of Gamrekelashvili *et al*^16^. and highlight the importance of the Notch pathway in mediating immunomodulatory mechanisms.

How does Tg6F induce the Notch pathway? Based on the work of Briot A *et al*.^19^, we believe the increased Notch pathway expression in Tg6F treated mice (Figs 3–5) is most likely due to the Tg6F-mediated reduction in oxidized phospholipids recognized by E06.

How does Tg6F treatment inhibit osteopontin (Spp1) protein? Watson *et al*.^21^ reported that 25-OHC induces osteopontin (Spp1). Our laboratory recently demonstrated that Tg6F inhibited CH25H, which is the enzyme responsible for the synthesis of 25-OHC, in the small intestine of LDLR null mice fed a Western Diet^22^. Here, we show that Tg6F treatment reduced the levels of CH25H in jejunum (Fig. 7d) and reduced its product 25-OHC in jejunum (Fig. 7e) in CT-26 cell injected BALB/c mice receiving a normal chow diet. Consistent with the findings of Watson *et al*.^21^, we found that Tg6F treatment reduced the level of *Spp1* expression in the small intestine (Fig. 7b), in the plasma (Fig. 7c) and the lungs (Fig. 7a). Since MDSC fate and function are known to be mediated by Spp1^17,18^, we also think it likely that the reduction in Spp1 levels is at least in part responsible for the Tg6F-mediated reduction in MDSCs in small intestine and lung. Figure 8c provides a schematic representation of our hypothesis.

The data reported here further emphasize the importance of the small intestine in modulating pathological processes that occur far from the small intestine. The data reported here also suggest that exploiting the intestine-lung axis using oral apoA-I mimetic peptides may have significant therapeutic potential.

## Materials and Methods

### Materials

Transgenic tomatoes expressing the 6F peptide or control tomatoes expressing a marker protein (β-glucuronidase) were grown and freeze-dried as described previously^14^. All other materials were purchased from sources previously described^28^.

### Mice

Female BALB/c mice, 6-weeks of age, or female C57BL/6J mice, 2-months of age were purchased from Jackson Laboratory and maintained on standard mouse chow (Teklad, Harlan, catalog no. T7013M15, Indianapolis, Indiana, USA). The animal protocols used in this study were approved by the Animal Research Committee at UCLA and the methods were carried out in accordance with the approved guidelines.

### Tumor cells

The CT26 cell line derived from N-nitroso-N-methyl urethane-induced mouse colon carcinoma of BALB/c origin was purchased from the American Type Culture Collection (ATCC: CRL-2639). The murine Lewis lung carcinoma cell line (3LL) was also obtained from ATCC (CRL-1642).

### Preparation of diets

Concentrates of the freeze-dried transgenic tomatoes expressing the 6F peptide (Tg6F) or the control marker protein (EV) were prepared and added to chow at 0.06% by weight as described previously^13,28^. The diets were packaged into 28 gram portions in aluminum foil and kept at −80 °C until use. The administration of the tomato concentrates began the day after the cancer cells were injected.

### Tumors

Female BALB/c mice 6 weeks of age were administered 2 × 10^4^ CT26 cells in 100uL of PBS *via* tail vein injection as described previously^6^. The day after injection, the mice were maintained on standard mouse chow or were switched to standard mouse chow containing 0.06% by weight of Tg6F. After 4 weeks the mice were subjected to a terminal bleed, and after sacrifice the lungs were harvested, weighed, and fixed with Bouin’s solution (Sigma, St. Louis, MO, USA), and the number of tumor nodules on the surface of the lungs was determined as previously described^6^. In other experiments, female C57BL/6J mice 2-months of age were administered 0.15 × 10^6^ 3LL cells via tail vein injection. The day after injection, the mice were maintained on standard mouse chow or were switched to standard mouse chow containing 0.06% by weight of the control tomato concentrate (EV) or the concentrate containing the 6F peptide (Tg6F). After one month, the lungs were harvested, weighed, fixed and the number of tumors on the surface of the lungs was determined.

### Harvesting of Jejunum and Preparation of Enterocytes

Mice were fasted overnight prior to the day of sacrifice. They were administered an overdose of isoflurane anesthesia, and perfused to remove blood prior to harvesting organs as described previously^14,28,51^. Enterocytes were prepared as previously described^22,28^.

### Gene Array Studies

Total RNA was extracted from lung and jejunum tissue and subjected to gene expression array analysis using NanoString nCounter PanCancer Mouse Pathway Panel (NanoString Technologies) according to the manufacturer’s protocol. The expression values were normalized using positive controls to eliminate platform-related variation, negative controls to eliminate background effect, and housekeepers to remove variation due to sample input. A total of 770 genes were included in the group comparisons. Linear models for microarray were built to compare groups regarding log_2_ expression values. A gene was considered significantly changed if p < 0.05 and if the change in expression for Control (chow) vs. Transgenic (Chow + Tg6F fed mice) was upregulated or downregulated 1.5-fold. Supplemental Fig. 2 shows a heat map of Notch family genes in jejunum.

### Protein Expression

Lung and jejunum tissue was lysed in RIPA lysis buffer (Catalog#8990, Thermofisher). Equal concentrations of the tissue were separated by SDS-PAGE gradient (10%) gel and then transferred onto nitrocellulose membranes and incubated overnight at 4 °C with primary antibodies (Dll1; ab84620. Abcam, dilution 1:1000, Dll4; ab7280, Abcam, dilution 1:1000, Notch1; #3608, Cell signaling, dilution 1:1000, Notch2; C651-56 DbHN-C, Developmental Studies Hybridoma Bank, dilution 1:1000), GAPDH (G9545, Sigma, dilution1: 20,000–40,000), SPP1; LS-C312596, Life Sciences, dilution 1:1000). The membranes were further incubated with species dependent HRP-conjugated secondary antibodies (1:10,000) at room temperature for 1 hour. Immuno-complexes were detected by enhanced chemiluminescence with Immobilon Western Chemiluminescent HRP Substrate (# WBKLS0500) and developed with X-Ray film. Supplementary Fig. 6 shows the whole immunoblots for Figs 4c,d and 7a.

### Dll4, E06, and cholesterol 25-hydroxylase (CH25H) immunohistochemistry (IHC)

Five to six mice (chosen at random) from each treatment group were used for IHC. Three to five segments of jejunum from each mouse were analyzed by the Immunohistochemistry Core in the Department of Pathology at David Geffen School of Medicine at UCLA. The jejunum segments were fixed in 10% neutral buffered formalin (pH 7.4) at physiological pressure at room temperature overnight. The fixed segments were thoroughly washed with distilled water and transferred to 70% ethanol followed by embedding in paraffin and sectioning. To detect E06, and CH25H, the slides were processed to remove paraffin, rinsed in PBS containing 0.05% Tween-20 (PBST), and then incubated at room temperature for 45 min with E06 antibody (Avanti Polar Lipids, catalog #330001 S) at a 1:50 dilution or anti-CH25H antibody (Bioss Antibodies, bs-6480R) at a dilution of 1:100. The slides were rinsed in PBST and the signals were amplified using VECTSTAIN Elite ABC kit (Vector Laboratories, catalog #PK-6100) following the recommended procedures. After a rinse with PBST, the slides were incubated with 3,3′-diaminobenzidine for visualization. The slides were washed in tap water and counterstained with Harris Hematoxylin, followed by dehydration in ethanol and mounting. Controls consisted of sections exposed to secondary-only antibodies. Photomicrographs of the sections were captured using an Olympus BX51 microscope and the application Q Capture 7.0 (Q Imaging, Inc.). Randomly selected fields were quantified for each sample and the ratio of the stain signal to the villus area of the jejunum was determined using Image Pro Plus 7 (Media Cybernetics). Supplementary Fig. 3 shows an example of IHC for CH25H in the villi of the jejunum.

### Method for immunofluorescence

For immunofluorescence imaging, tissue sections were deparaffinized and antigen retrieval was performed with boiling citrate buffer (10 mM in PBS, pH 6) for detection of Notch1 and Dll4. Primary antibodies used were anti-Notch1 (D1E11) rabbit monoclonal (1:200; Cell Signaling #3608), anti-Notch2 (1:100; C651-56 DbHN-C, Developmental Studies Hybridoma Bank), anti-Dll1 (1:100; Abcam #ab84620), and anti-Dll4 rabbit polyclonal (1:100; Abcam #ab7280) with fluorescently tagged secondary antibodies purchased from Invitrogen, applied at 1:400 dilutions. Images were captured with 20X or 40X objective on a Zeiss LSM880 confocal microscope with ZEN software (Carl Zeiss Microscopy) for acquisition. Image processing was performed using Imaris software (Bitplane).

### Flow cytometry

Mice were fasted overnight prior to the day of sacrifice. They were administered an overdose of isoflurane anesthesia, and perfused to remove blood prior to harvesting organs as described previously^14,28,51^. Immune cells were isolated from lung by lung lavage^21^ and from the lamina propria of the jejunum using a Miltenyi Biotech lamina propria dissociation kit (Catalog #130-097-410) as described^28^. Single cell suspensions were incubated with Zombie Aqua (Biolegend, Catalog # 423101) together with anti-CD45 (Biolegend, catalog#103146), anti-CD11b (Biolegend, Catalog#101211), anti-Ly6C (Biolegend, Catalog#128037), anti-Ly6G (Biolegend, catalog#127639), anti-CX3CR1(Biolegend, Catalog#149015). After 45 min, the cells were washed twice with fluorescence-activated cell sorting (FACS) buffer (PBS + 5% FBS). After a short spin, the cells were suspended in 300 μL of ice-cold PBS buffer and transferred to fresh tubes for FACS analysis. FACS was performed using a BD LSR Fortessa X-20 machine SORP version 8.0.1 in the Janis V. Giorgi Flow Cytometry Core Facility at UCLA. For analysis and computational compensation of the data, BD FACS Diva software was used. Ten thousand events of live cells were gated. Only live and singlet cells were chosen for analysis and gating (i.e., dead cells and aggregates were excluded).

### RNA Extraction

Mice were fasted overnight prior to the day of sacrifice. They were administered an overdose of isoflurane anesthesia, and perfused to remove blood prior to harvesting organs as described previously^14,51^. The jejunum and lungs were removed and total RNA was isolated with Ambion *mir*vana MiRNA kit (AM-1560). The fold change of gene expression was analyzed by RT-qPCR as described previously^51^.

### Determination of 25-OHC in jejunum by LC-MS/MS

Quantitation of 25-OHC in jejunum samples that had been stored at −80 °C was carried out as described previously^22^.

### Statistical Analysis

Statistical analyses were performed initially by ANOVA. After determining that statistically significant differences were present by ANOVA, further comparisons were made by unpaired two-tailed *t*-test. All statistical analyses were performed using Graph-Pad Prism version 5.03 or version 7.03 (GraphPad Software, San Diego, CA). Statistical significance was considered achieved if *p* < 0.05.

## Electronic supplementary material

Supplementary figures
