# Supplementary material for: Treating the Intestine with Oral ApoA-I Mimetic Tg6F Reduces Tumor Burden in Mouse Models of Metastatic Lung Cancer
Source: Sci Rep. 2018 Jun 13;8:9032. doi: 10.1038/s41598-018-26755-0 (PMC5998131; doi:10.1038/s41598-018-26755-0)
Supplement: Supplementary file 1 — Supplementary figures [file 41598_2018_26755_MOESM1_ESM.pdf]

Treating the Intestine with Oral ApoA-I Mimetic Tg6F Reduces Tumor Burden in Mouse Models of Metastatic Lung Cancer

Arnab Chattopadhyay*, Xinying Yang§, Pallavi Mukherjee*, Dawoud Sulaiman*δ, Hannah R. Fogelman*, Victor Grijalva*, Steven Dubinett*, Tonya C. Wasler*, Manash K Paul*, Ramin Salehi-Rad*, Julia J. Mack¶, M. Luisa Iruela-Arispe¶, Mohamad Navab*, Alan M. Fogelman*, and Srinivasa T. Reddy*†§δ€

Departments of Medicine*, Molecular and Medical Pharmacology†, Obstetrics and Gynecology§, Molecular Toxicology Interdepartmental Degree Programδ, David Geffen School of Medicine at UCLA, Department of Molecular, Cell and Developmental Biology, College of Letters and Science, University of California, Los Angeles¶, Los Angeles, CA 90095-1736

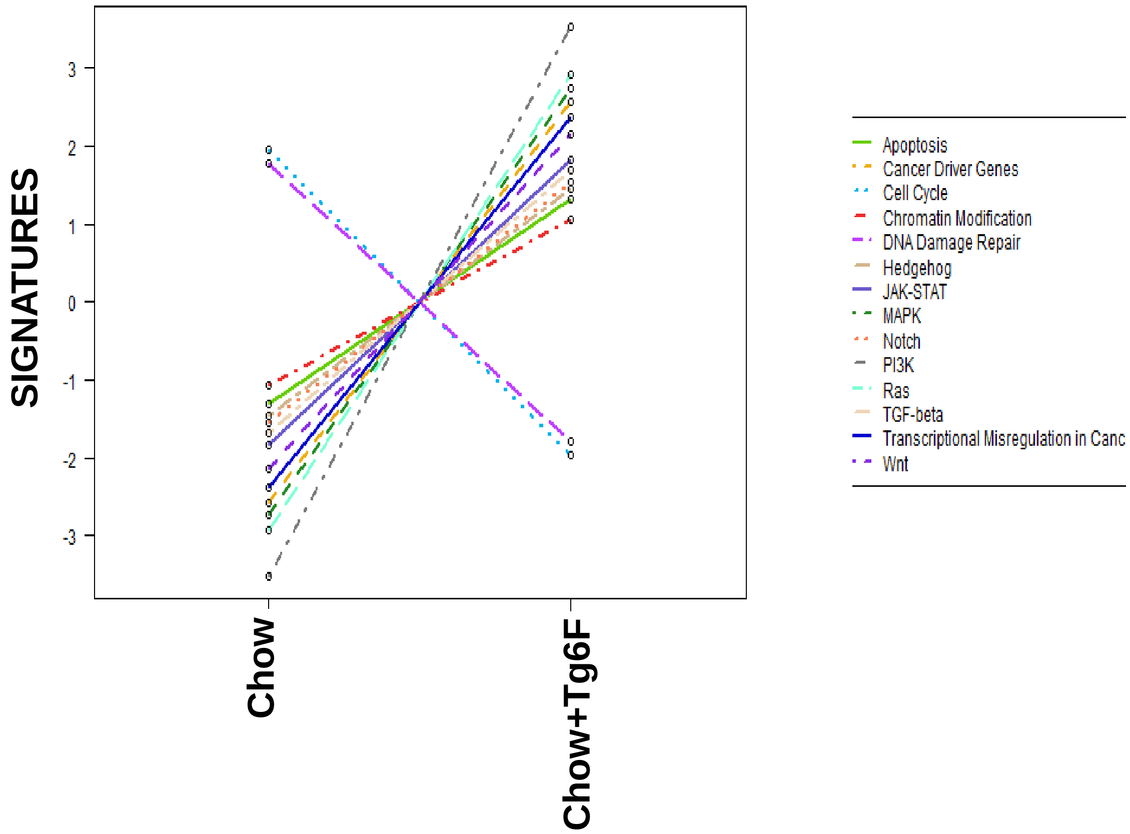

Supplemental Figure 1. Differentially expressed genes in NanoString nCounter PanCancer mouse pathway Panel. Trend plot of signature (770 genes from 13 cancer-associated canonical pathways) vs. Category (Chow: Chow + Tg6F fed mice) in lung. Most of the genes were upregulated in mice receiving Tg6F, compared to control mice where most genes were downregulated, only the cell cycle & DNA damage repair related genes were higher in the control mice.

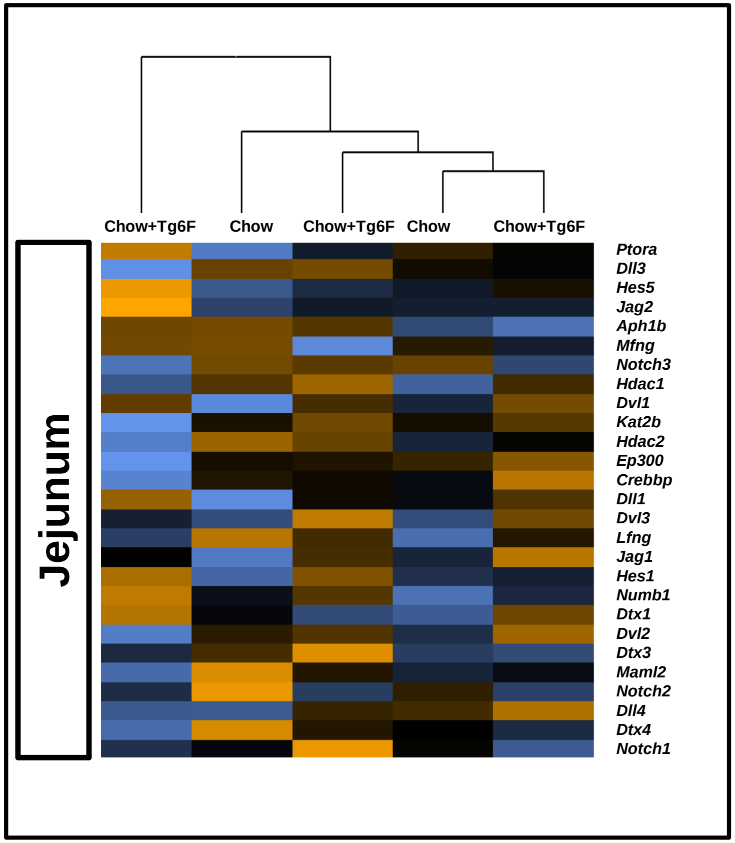

Supplemental Figure 2*: Gene array analysis and expression of Notch pathway genes.* Heat map of Notch family genes in Jejunum tissue obtained from control and Tg6F groups.

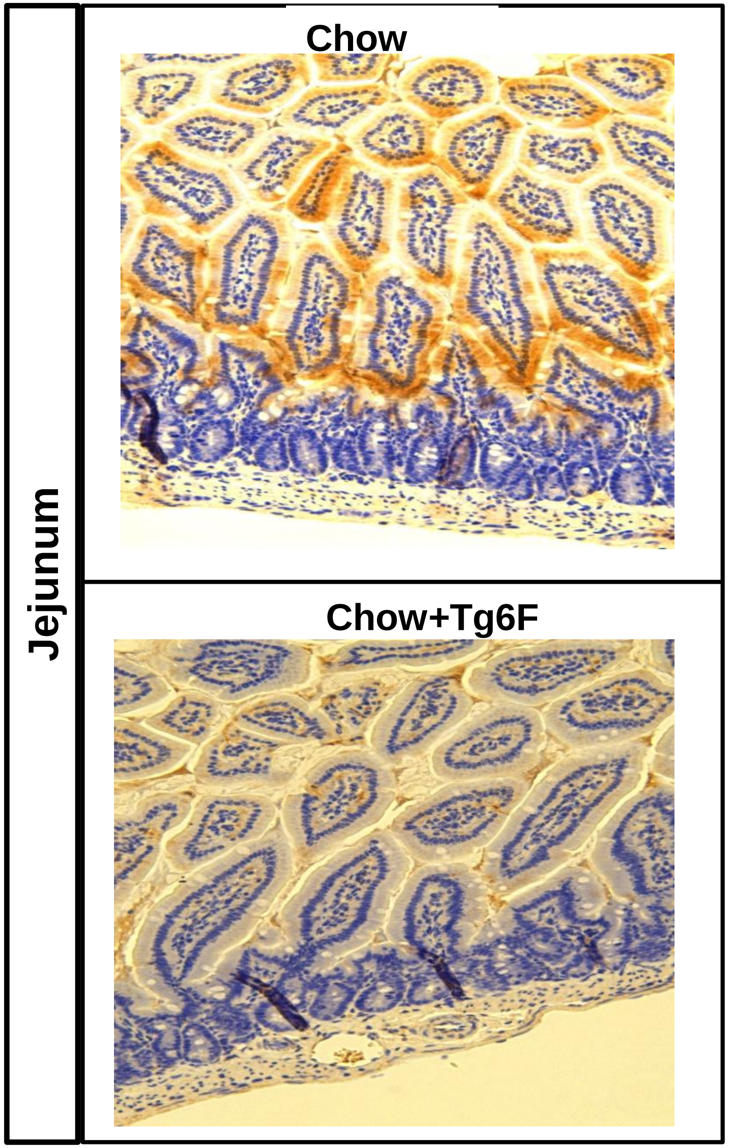

Supplemental Figure 3: Cholesterol 25-hydroxylase (CH25H) protein expression in the villi of the jejunum was quantified by immunohistochemistry as described in Materials and Methods. Representative photomicrograph of a section of jejunum stained for CH25H (brown).

Jejunum Lamina Propria Patrolling Monocytes

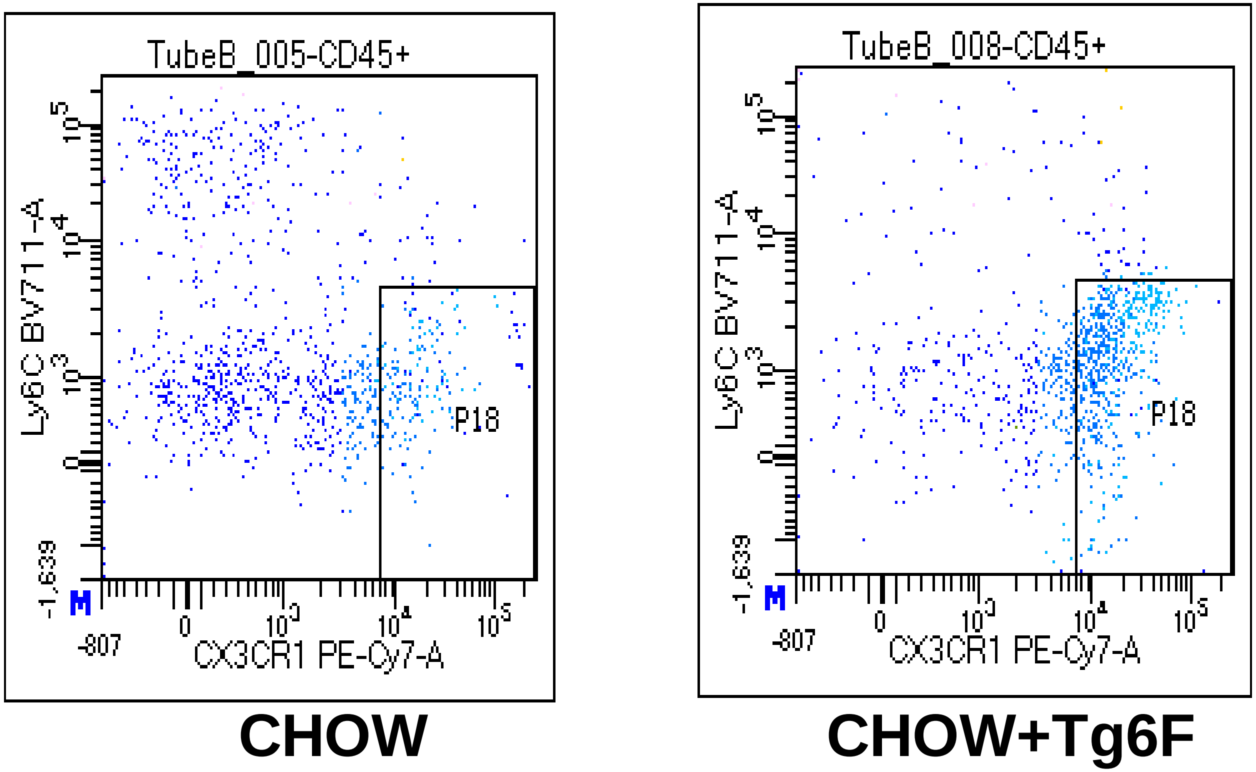

Supplemental Figure 4. An example of flow cytometry data for patrolling monocytes isolated from the lamina propria of the jejunum.

Jejunum Lamina Propria Myeloid Derived Suppressor Cells

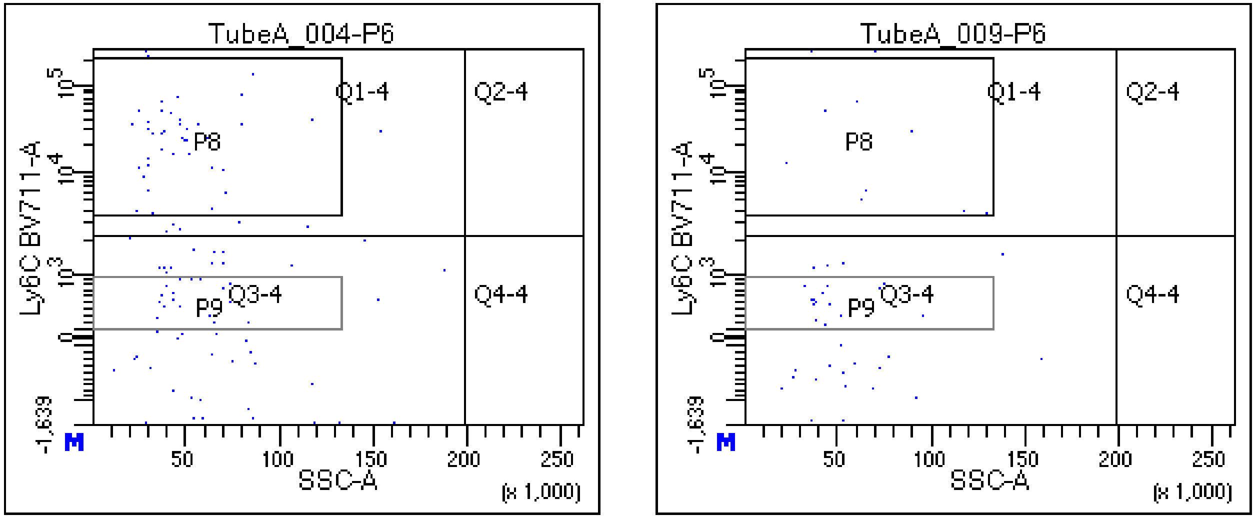

CHOW **CHOW+Tg6F**

Supplemental Figure 5. An example of flow cytometry data for myeloid derived suppressor cells isolated from the lamina propria of the jejunum.

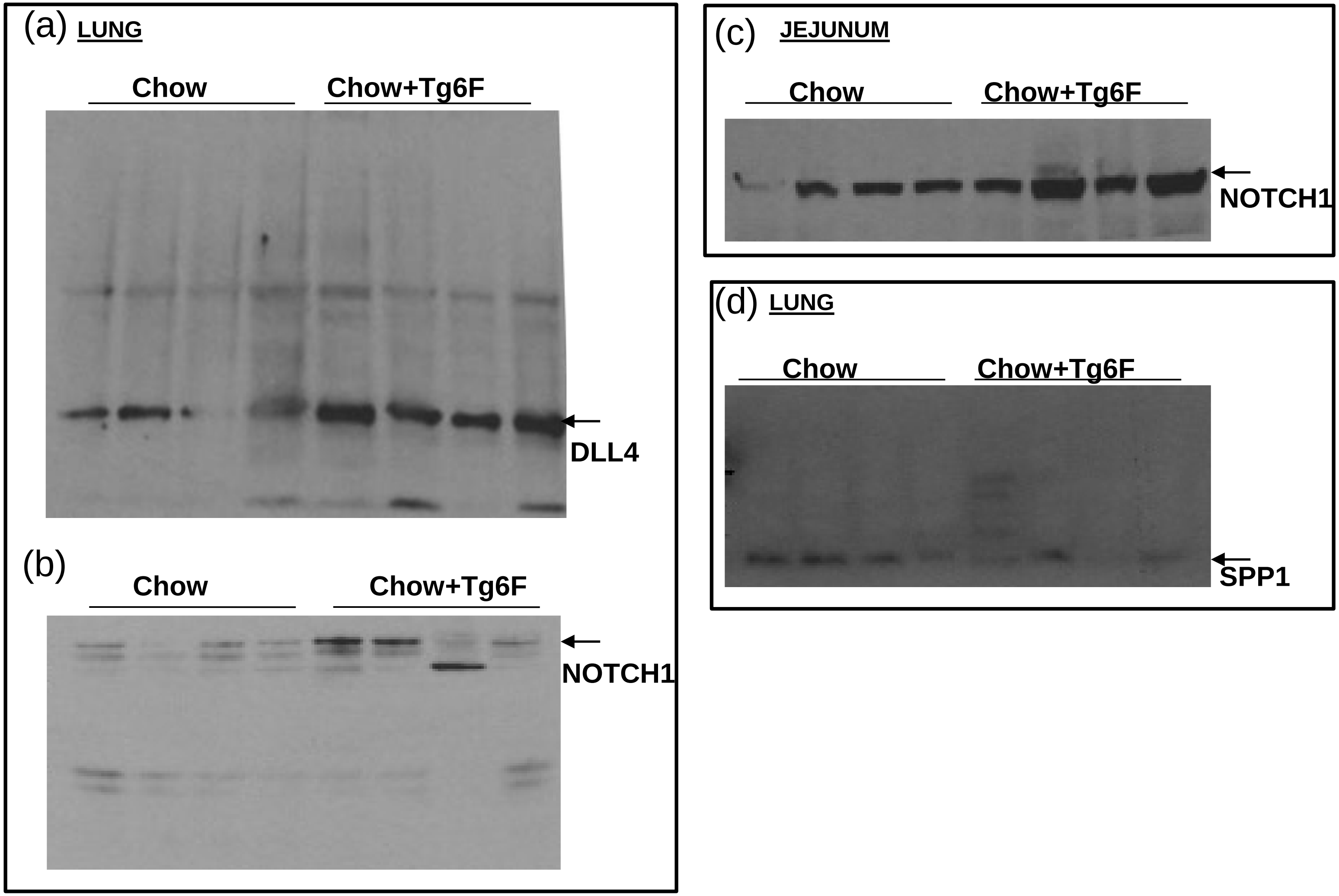

Supplemental Figure 6. Supplementary information for the Immunoblot in Figure 4 c, d and Figure 7a. The whole Immunoblot, (a and b) protein expression of Dll4 and Notch1 in lung, (c) Protein expression of Notch1 in jejunum, (d) Protein expression in SPP1 in lung.
